# Dietary Intake in Population with Metabolic Syndrome: Is the Prevalence of Inadequate Intake Influenced by Geographical Area? Cross-Sectional Analysis from PREDIMED-Plus Study

**DOI:** 10.3390/nu10111661

**Published:** 2018-11-04

**Authors:** Naomi Cano-Ibáñez, Aurora Bueno-Cavanillas, Miguel A. Martínez-González, Dolores Corella, Jordi Salas-Salvadó, M. Dolors Zomeño, Manoli García-de-la-Hera, Dora Romaguera, J. Alfredo Martínez, F. Javier Barón-López, Antonio García-Ríos, Ramón Estruch, Laura García-Molina, Ángel Alonso Gómez, Josep A. Tur, Francisco Tinahones, Lluis Serra-Majem, Naiara Cubelos-Fernández, José Lapetra, Clotilde Vázquez, Xavier Pintó, Josep Vidal, Lidia Daimiel, José Juan Gaforio, Pilar Matía, Emilio Ros, Javier Diez-Espino, Rebeca Fernández-Carrión, Josep Basora, Montse Fitó, Juan Manuel Zazo, Antoni Colom, Estefanía Toledo, Andrés Díaz-López, Miguel Ángel Muñoz, Miguel Ruiz-Canela, Alfredo Gea

**Affiliations:** 1 Department of Preventive Medicine and Public Health, University of Granada, 18011 Granada, Spain; abueno@ugr.es (A.B.-C.); lgarmol@ugr.es (L.G.-M.); 2 CIBER of Epidemiology and Public Health (CIBERESP), Carlos III Institute of Health, 28029 Madrid, Spain; manoli@umh.es (M.G.-d.-l.-H.); jgaforio@ujaen.es (J.J.G.); juanmazazo@gmail.com (J.M.Z.); 3 Department of Preventive Medicine and Public Health, Medical School, University of Navarre, 31008 Pamplona, Spain; mamartinez@unav.es (M.A.M.-G.); javierdiezesp@ono.com (J.D.-E.); etoledo@unav.es (E.T.); mcanela@unav.es (M.R.-C.); ageas@unav.es (A.G.); 4 Department of Nutrition, Harvard T.H. Chan School of Public Health, Boston, MA 02115, USA; 5 CIBER Physiopathology of Obesity and Nutrition (CIBEROBN), Carlos III Institute of Health, 28029 Madrid, Spain; dolores.corella@uv.es (D.C.); jordi.salas@urv.cat (J.S.-S.); doraromaguera@yahoo.es (D.R.); jalfmtz@unav.es (J.A.M.); baron@uma.es (F.J.B.-L.); angarios2004@yahoo.es (A.G.-R.); restruch@clinic.ub.es (R.E.); angelmago13@gmail.com (Á.A.G.); pep.tur@uib.es (J.A.T.); fjtinahones@hotmail.com (F.T.); lluis.serra@ulpgc.es (L.S.-M.); jlapetra@ono.com (J.L.); clotilde.vazquez@fjd.es (C.V.); xpinto@bellvitgehospital.cat (X.P.); eros@clinic.ub.es (E.R.); rebeca.fernandez@uv.es (R.F.-C.); jbasora.tarte.ics@gencat.cat (J.B.); mfito@imim.es (M.F.); antonicolom@gmail.com (A.C.); andres.diaz@urv.cat (A.D.-L.); 6 Department of Preventive Medicine, University of Valencia, 46010 Valencia, Spain; 7 Human Nutrition Unit, Biochemistry and Biotechnology Department, IISPV, Universitat Rovira i Virgili, 43002 Reus, Spain; 8 Unit of Cardiovascular Risk and Nutrition, Institut Hospital del mar de Investigaciones Médicas Municipal d’Investigació Médica (IMIM), 08003 Barcelona, Spain; mariadoloreszf@blanquerna.edu; 9 Human Nutrition Unit, Blanquerna-Ramon Llull University, 08001 Barcelona, Spain; 10 Miguel Hernández University, ISABIAL-FISABIO, 03202 Alicante, Spain; 11 Health Research Institute of the Balearic Islands (IdISBa), 07120 Palma de Mallorca, Spain; 12 Department of Nutrition, Food Sciences and Physiology, Center for Nutrition Research, University of Navarra, 31008 Pamplona, Spain; 13 Nutritional Genomics and Epigenomics Group, IMDEA Food, CEI UAM + CSIC, 28049 Madrid, Spain; lidia.daimiel@imdea.org; 14 Department of Public Health, University of Málaga-IBIMA, 29016 Málaga, Spain; 15 Lipids and Atherosclerosis Unit, Department of Internal Medicine, Maimonides Biomedical Research Institute of Córdoba (IMIBIC), Reina Sofía University Hospital, University of Córdoba, 14004 Córdoba, Spain; 16 Department of Internal Medicine, IDIBAPS, Hospital Clinic, University of Barcelona, 08036 Barcelona, Spain; 17 Department of Cardiology, OSI ARABA, University Hospital Araba, University of the Basque Country (UPV/EHU), 48940 Vitoria-Gasteiz, Spain; 18 Research Group on Community Nutrition & Oxidative Stress, University of Balearic Islands, 07122 Palma de Mallorca, Spain; 19 Virgen de la Victoria Hospital, Department of Endocrinology, University of Málaga, 29010 Málaga, Spain; 20 Institute for Biomedical Research, University of Las Palmas de Gran Canaria, 35016 Las Palmas, Spain; 21 Institute of Biomedicine (IBIOMED), University of León, 24071 León, Spain; n.cubelos.fernandez@gmail.com; 22 Department of Family Medicine, Research Unit, Distrito Sanitario Atención Primaria, 41013 Sevilla, Spain; 23 Department of Endocrinology, Fundación Jiménez-Díaz, 28040 Madrid, Spain; 24 Lipids and Vascular Risk Unit, Internal Medicine, Hospital Universitario de Bellvitge, Hospitalet de Llobregat, 08907 Barcelona, Spain; 25 Department of Endocrinology, IDIBAPS, Hospital Clinic, University of Barcelona, 08036 Barcelona, Spain; jovidal@clinic.cat; 26 CIBER Diabetes y Enfermedades Metabólicas (CIBERDEM), Instituto de Salud Carlos III (ISCIII), 28029 Madrid, Spain; 27 Centro de Estudios Avanzados en Olivar y Aceites de Oliva, University of Jaén, 23071 Jaén, Spain; 28 Department of Endocrinology and Nutrition, Instituto de Investigación Sanitaria Hospital Clínico San Carlos (IdISSC), 28040 Madrid, Spain; pilar.matia@gmail.com; 29 Lipid Clinic, Department of Endocrinology and Nutrition, Institut d’Investigació Biomédiques August Pi Sunyer (IDIBAPS), Hospital Clinic, 08036 Barcelona, Spain; 30 Servicio Navarro de Salud, Osasunbidea, 31002 Pamplona, Spain; 31 Primary Care Division of Barcelona, Institut Català de la Salud-IDIAP Jordi Gol, 08007 Barcelona, Spain; mamunoz.bcn.ics@gencat.cat

**Keywords:** dietary intake, PREDIMED-Plus study, metabolic syndrome, place of residence, geographical area, nutrient adequacy

## Abstract

Inadequate diet influences chronic diseases such as cardiovascular disease (CVD), the leading cause of death in Spain. CVD figures vary from one geographical region to another; this could be associated with different food choices. Our aim was to analyse the influence of geographical area on nutrient intakes among the Spanish adult population with Metabolic Syndrome (MetS). We analysed cross-sectional baseline data from the PREDIMED-Plus study: 6646 Spanish adults, aged 55–75 years, with overweight/obesity and MetS in four geographical areas. A validated 143-item Food Frequency Questionnaire (FFQ) was used to assess energy and nutrient intakes. The prevalence of inadequate nutrient intake was estimated according to Dietary Reference Intakes (DRIs). Multivariable-adjusted logistic regression was used to assess the relationship between geographical area (North, Central, East and South areas) and inadequate nutrient intake. People in the North area consumed significantly lower amounts of vegetables and fish but more sugar and alcohol (*p* < 0.001) than other areas. Dietary fibre, vitamin A, E, calcium and magnesium intakes were all lower among men of North area than in the other areas (*p* < 0.001). Sex (women), non-smoker and physical activity were also associated to adequate nutrient intake. Geographical area influences nutrient intakes. Its effect on dietary quality should be taken into account when planning food policies.

## 1. Introduction

Over the last decades, overweight and obesity have increased in most regions and countries worldwide [1]. A similar situation has been described in Spain. The prevalence of overweight has increased in the last 20 years with obesity figures now twice what they were twenty years ago and they have especially increased in the aged adult Spanish population [2]. The global burden of obesity and overweight can be explained by the increase in the consumption of diets with high energy density and low nutritional value, a consequence of the acquisition of Westernized dietary patterns [3]. 

A healthy dietary pattern which includes low amounts of saturated fat, salt and refined carbohydrates and promotes the consumption of high amounts of fruit, vegetables and whole grains have proved not only to reduce the risk of overweight and obesity but also to have a direct effect on chronic diseases incidence and prognosis which affect global health [4]. 

The traditional Mediterranean diet (MedDiet) described in the 1950s and 1960s [5], is characterized by frugality or moderation on food consumption, a high intake of vegetables, legumes, fruits and nuts, unrefined cereals, fish and olive oil and a low intake of saturated lipids, dairy products and red meat [6]. MedDiet prevents diseases that especially affect the aged population, such as neurodegenerative diseases, diabetes mellitus and some types of cancer [7,8,9,10]. Furthermore, MedDiet has also been associated with protection against some of the leading causes of morbidity and mortality worldwide, in particular cardiovascular disease (CVD) and metabolic syndrome (MetS) [11,12]. MetS is a well-described condition in the causative pathway of cardiovascular disease attributable to clustering factors that includes central obesity, insulin resistance, dyslipidaemia and hypertension [13]. 

Moreover, accumulated evidence suggests that the elevated overweight and obesity rates around the world are linked to MetS, coexisting with nutritional deficits in the adult population [14]. In this context, it is important to note that an increase in adherence to MedDiet is associated with better diet quality and lower prevalence of deficient intake of certain micronutrients. That is, the MedDiet has demonstrated supportive effects, not only on the protection against chronic diseases but also on the aged population’s nutritional status [15]. 

The nutritional status of the aged population is an important public health issue; adequate dietary intake plays an important role in the healthy aging process. For a variety of reasons, that include economic, social and physiologic changes, aged adults are at risk of low dietary quality. Aging is accompanied by an increased need in several nutrients, such as vitamins and minerals, whereas the overall caloric requirements decline [16,17]. In this sense, the consumption of a high-quality diet such as MedDiet is essential to reduce malnutrition figures in older adults. Although some studies suggest that the adherence to MedDiet in Spain is greater than in other Mediterranean countries [18], recently published data on dietary patterns in Spanish adults indicated that adherence to the MedDiet has substantially declined [19]. 

In fact, besides age, sex or cultural level, the adherence to the MedDiet in Spain varies significantly among geographical areas. As an example, the Southern regions of Spain had the lowest score for adherence to the MedDiet in comparison with Northern regions [20,21], even though other authors have reported that place of residence has limited influence on dietary intake in the adult and senior adult population [22]. Therefore, differences in dietary intake do not only differ among Mediterranean countries but also within the same country, most likely because each area has its own gastronomic culture influenced by particular socioeconomic and cultural factors related to food choices and other determinants of dietary intake [20]: This could be responsible for the North-South cardiovascular mortality gradient in Spain [23].

To our knowledge little is known about dietary intake and inadequate nutrient intake among the older Spanish adults with MetS from different geographical areas of Spain. Our aim was to analyse the influence of geographical area on nutrient intakes among the Spanish adult population with MetS.

## 2. Materials and Methods 

### 2.1. Study Design

This research represents a cross-sectional study on baseline data of the PREDIMED-Plus trial study. PREDIMED-Plus study is a 6-year Spanish multicentre ongoing randomized, parallel-group clinical trial testing the effect on CVD morbimortality of an intensive weight-loss intervention program based on an energy-restricted traditional MedDiet, physical activity promotion and behavioural support, in comparison with a usual care intervention only with energy-unrestricted Mediterranean diet (control group). The participant recruitment methods and data collection are fully described in a publication by Martinez-Gonzalez MA et al. [24] and at http://predimedplus.com/. The PREDIMED-Plus study protocol was approved according to the ethical standards of the Declaration of Helsinki by the respective Institutional Review Board (IRB) in each centre and registered at the International Standard Randomized Controlled Trial (ISRCT), with number 89898870. For more information, http://www.isrctn.com/ISRCTN89898870. Registration date: 24 July 2014. Informed consent was obtained from all individual participants included in the study. 

### 2.2. Participants and Data Collection Procedures

**Figure 1:**
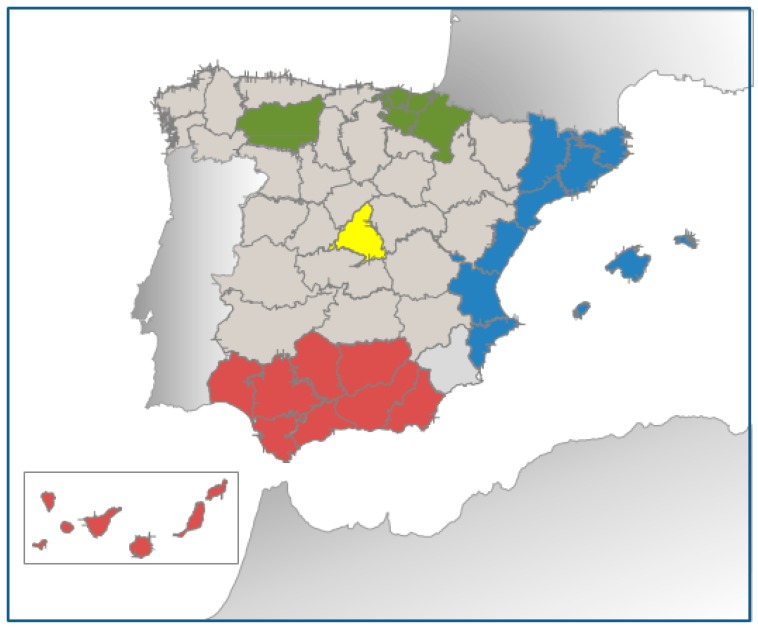
Map of Spain with the geographical areas analysed.

The population of the study consisted of a total sample of 6874 aged adults of both genders that were selected and randomized in 23 centres of recruitment, including different universities, hospitals and Spanish research institutes, of the PREDIMED-Plus clinical trial from 14 Spanish provinces (Alicante, Barcelona, Cordoba, Granada, Jaen, Las Palmas de Gran Canaria, Leon, Madrid, Malaga, Mallorca, Navarra, Sevilla, Valencia and Vitoria), from October 2013 to November 2016. These centres recruited participants from Primary Care Health clinics belonging to the National Health System. Potential eligible participants were adult men aged between 55 and 75 years and women between 60 and 75 years with a body mass index between ≥27 and <40 kg/m^2^ and meeting at least 3 criteria for MetS [13] but with no previous CVD. Participants were organized into 4 different areas (Figure 1) according to geographical area of residence (Nielsen areas) [22]. However, some regions have been grouped with contiguous geographical area, in the same way that other authors have described previously [20], due to the small sample size. North area (green-North) includes the northern areas: Leon, Navarra and the Basque Country. Central area (yellow-Central) comprises the central area of the country: Madrid. East area (blue-East) is composed of the areas located in eastern Spain: Cataluña, Valencia and Balearic Islands. Finally, South area (red-South) covers the south of Spain (Andalucía) and Canary Islands.

**Figure 2:**
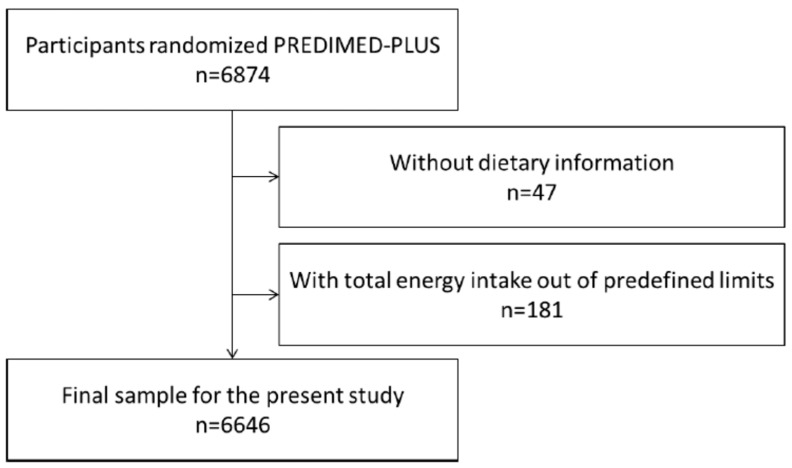
Flow-chart of participants.

For the current analysis, 228 participants were excluded (Figure 2) because they reported values for total energy intake outside of the predefined limits (<800 kcal/day or >4000 kcal/day for men); (<500 kcal/day or >3500 kcal)/day for women). These limits were set in accordance with recommendations by Willett in Nutritional Epidemiology [25]. In addition, 47 participants were excluded because they did not complete the Food Frequency Questionnaire (FFQ). After applying these exclusion criteria, a final sample of 6646 participants was analysed.

### 2.3. Assessment of Dietary Intake

Trained nutritionists collected baseline food intake data in their baseline interview using the PREDIMED FFQ, which has been previously validated in Spanish subjects [26,27]. The FFQ included 143 food items, arranged into 11 food groups, taking into account the similarities of their nutrient profiles (vegetables, fruits, legumes, cereals, milk and dairy products, meat and meat products, fish and seafood, olive oil, nuts, sugars and sweets and eggs). The FFQ includes 9 possible answers for consumption frequency ranging from never/seldom to >6 servings/day. The indicated frequencies of consumption were converted to intakes per day and multiplied by the weight of the portion size indicated by the participant and expressed as grams per day [28]. Nutrient information was derived using Spanish food composition tables [29,30]. Subjects were asked about MedDiet adherence via a 17-item screening questionnaire, which contains modifications to a previously validated 14 items questionnaire [31], used to evaluate compliance with the intervention and was also a key element to guide the motivation interviews during the study follow-up. Compliance with each of the 17 items relating to characteristic food habits was scored with 1 point and 0 points for non-adherence. Therefore, the total score range was 0–17, with 0 meaning no adherence and 17 meaning maximum adherence. Adherence to MedDiet was categorised in tertiles, as low level of adherence (1st tertile, ≤7 points), medium (2nd tertile, 8–10 points) or high level of adherence (3rd tertile, ≥11 points). Dietary intake of total energy, total fat and fatty acids: monounsaturated (MUFAs), polyunsaturated (PUFAs) and saturated (SFAs), proteins and carbohydrates were estimated. Dietary fibre and alcohol intake were also measured. Inadequate nutrient intake of dietary fibre and vitamins A, D, E, B_12_, B_6_, B_9_ and minerals such as calcium, magnesium and phosphorus were analysed according to the Dietary Reference Intakes (DRIs) for the American population for each participant [32]. DRIs is the general term for a set of reference values used to plan and assess nutrient intakes of healthy people. These values vary by age and sex and include the Recommended Dietary Allowance (RDA): average daily level of intake sufficient to meet the nutrient requirements of nearly all (97–98%) healthy people, Adequate Intake (AI): established when evidence is insufficient to develop an RDA and is set at a level assumed to ensure nutritional adequacy and Tolerable Upper Intake Level (UL): maximum daily intake unlikely to cause adverse health effects [33]. Intake levels above DRI imply a low likelihood of inadequate intake. To decrease possible measurement errors derived from FFQ, we estimated the proportion of individuals with intakes below two thirds (2/3) of the DRIs, correcting the possible bias introduced by the FFQ and assuming in any case that the actual inadequate intake should be higher than the figures which were estimated [34]. Furthermore, we have estimated the inadequate intake according to the European Food Safety Agency (EFSA) average requirements (AR), taking as reference AI when AR were not available [35]. We also studied the proportion of participants with inadequate intake of 3 or more out of 6 nutrients. Results were based on dietary intake data only, excluding intake of supplements. 

### 2.4. Assessment of Non-Dietary Intake

During the baseline visit, trained staff of PREDIMED-Plus trial documented information using a baseline questionnaire on sociodemographic data and lifestyle behaviours. The variables included were sex, age (55–70 years and >70 years), geographical area (North, Central, East or South area), education level (primary level, secondary level and tertiary level that includes university studies), civil status (married, widowed, divorced/singled or others) and whether they lived alone or not. Other lifestyle variables such as smoking habits, alcohol intake (grams per day) and physical activity were taken into account. Physical activity information was gathered using the validated Spanish version of the Minnesota questionnaire [36,37].

### 2.5. Statistical Analysis

We used the PREDIMED-Plus baseline database generated in August 2017. Qualitative variables were analysed through their frequency distribution, whereas quantitative variables were expressed as means and standard deviations (SD). Pearson’s chi-square tests and Analysis of Variance (ANOVA) (for categorical and continuous variables, respectively) were used to assess differences in baseline characteristics of participants according to geographical area. Also, ANCOVA was used to estimate dietary intakes, adherence to MedDiet and nutrient profiles according to geographical area adjusted for sex and age. The significance level was set at 5%. The main outcome of this analysis was inadequate intake of micronutrients, defined for each nutrient as a daily intake below 2/3 of DRIs and set at 95% confidence interval (CI). We also repeated the analysis defining inadequate intake according to AR/AI proposed by EFSA. Geographical area was the principal independent variable. The associations between geographical area and inadequate intakes were summarized using odds ratios and 95% CI obtained via multiple-adjusted logistic regression models. The logistic regression model was adjusted for sex, age (55–70 years and >70 years), adherence to MedDiet (low, medium and high adherence), total energy intake, smoking habits (current, former or never smoker), physical activity (less active, moderately active and active), living alone, diabetic status and educational level (primary, secondary and tertiary). A logistic regression model was used to examine the associations between the place of residence and inadequate nutrient intake (defined as deficient intake for 3 or more nutrients) adjusted for the same potential confounding factors mentioned above.

Vitamins B_6_, B_12_ and phosphorus intakes were excluded from the logistic regression model, because a binary test showed these to be asymmetrically distributed and also a low proportion of subjects presented inadequate intake of these micronutrients. In addition, the intake of vitamin D was also excluded from the statistical analysis because it was deficient in a high proportion of the population (over 80%). All analysis were cross-sectional and performed using Stata (12.0, StataCorp LP, College Station, TX, USA).

## 3. Results

### 3.1. Baseline Characteristics of the Population and Food Intake

The baseline characteristics of participants and the food group intake in grams per day by geographical area are shown in Table 1. Mean patient age was 65.0 ± 4.9 years and 51.6% (*n* = 3431) of participants were male. When baseline characteristics were compared according to the four geographical areas included, differences were found in age, smoking habits, physical activity, cultural level and civil status. Regarding physical activity, 21.3% reported they were active, while 59.8% did not. Central area had the highest rates of active population (37.0%). The majority of the population had a primary educational level (48.3%, primary school) but, differences were evident among the 4 areas. The highest percentage of participants with only primary school (54.9%) was observed in South area. 

### 3.2. Description of Food Intake, Adherence to MedDiet and Nutrient Profiles in the Different Geographical Areas Analysed

Evaluation of food group intake, adherence to MedDiet and nutrient profiles among geographical areas of Spain adjusted for age and sex are shown in Table 2. According to the geographical area, the estimated adjusted mean intakes of fruits, sugar and sweets, olive oil, cereals and eggs were significantly higher among those participants living in North area than among those living in the other areas (*p* < 0.001). Whereas the intake of vegetables, nuts, legumes, fish and seafood were the lowest compared with the others geographical territories analysed (*p* < 0.001). The evaluation of food intake by sex and age among the geographical areas is shown in the Supplementary Materials (Table S1). In order to evaluate differences in MedDiet adherence across the geographical areas, we compared the mean score. The overall adherence score was 8.5 ± 2.7 (data not shown). According to geographical area, the East area showed the worst mean score of adherence, which was significantly lower than the mean score obtained in Central and South areas. The South area showed the lowest total energy intake, although, the contribution of healthy fats such as MUFAs and PUFAs was higher compared to the other geographical areas. Alcohol intake was higher in North area. The adherence to MedDiet and nutrients profile among areas stratified by sex and age is presented in Supplementary Table S2. Mean adherence to MedDiet increased in aged population (more than 70 years) in both sexes, being higher in women than in men. Total energy intake decreased with age in both sexes. Alcohol intake was higher in men than in women independently of age or place of residence, being the highest in the North area.

### 3.3. Evaluation of Inadequate Nutrient Intake According to Recommended Intakes

Table 3 shows the proportion of participants who showed nutrient intake below 2/3 of DRIs by geographical area. An inadequate intake of dietary fibre, vitamins A, B_9_, E and calcium was common in all the analysed groups of participants. Comparing the inadequate intakes by sex, men showed a higher prevalence than women of inadequate intake for most of the nutrients analysed. For the majority of nutrients, the prevalence of inadequate intake increased in women in older ages but not in men, except for calcium. In accordance to geographical areas, the North area presented the highest prevalence of inadequate intake for most nutrients analysed, except for vitamin B_9_ and dietary fibre, while East area presented the lowest prevalence of deficient intake for all the micronutrients analysed but differences are statistically significant only among the youngest participants and only for vitamins A, D and E. These results are similar if the EFSA recommendations are used (Table S3).

### 3.4. Assessment of the Influence of the Geographical Area on Inadequate Nutrient Intake

We examined the possible effect of geographical area on the inadequate intake of nutrients (Table 4), with North area as the reference category. The unadjusted logistic regression model revealed that the geographical area influenced the inadequate intake for all the nutrients analysed except for dietary fibre. In this sense, not living in the North area was significantly associated with a lower likelihood of inadequate intake of vitamin A, E, calcium and magnesium (*p* < 0.001). However, for vitamin B_9_, the East and the South areas had a higher probability of inadequate intake in comparison with the Northern reference area (OR = 1.19 and 1.31 respectively, *p* < 0.001). When we used the EFSA dietary recommendations, we obtained very similar results (Table S4). The influence of demographics (age, sex and place of residence) and other socioeconomic and lifestyle variables on inadequate nutrient intake according to the DRIs criteria is presented in the Supplementary Materials (Table S5). Inadequate nutrient intake was influenced by geographical area and sex. It was also inversely associated with better adherence to the MedDiet, total energy intake, not diabetic, being moderately active and being a non-smoker (*p* < 0.001). The Supplementary Table S6, shows similar associations according to EFSA criteria for all nutrients except for calcium.

### 3.5. Multivariable Logistic Regression Model for Inadequate Intake of 3 or More out 6 Micronutrients According to Place of Residence

Table 5 shows the result from the logistic regression model fitted using inadequate intake of 3 or more out of 6 micronutrients as the dependent variable. In the North area, 19.0% of participants showed inadequate intake for 3 or more. Dwellers of Central, East and South areas had a lower probability of inadequate intake for 3 or more micronutrients (*p* < 0.05). When we used AR/AI provided by the EFSA, we obtained higher figures (Table S7), reaching 40.8% of participants with inadequate intake in the North area.

Results are expressed as OR and 95% CI for the inadequate intake of ≥3 micronutrients as categorical variables according to geographical area. The model has been adjusted for sex, age, smoking habits, physical activity, educational status, diabetic status, living alone, total energy intake and adherence to MedDiet.

## 4. Discussion

Our results show significant differences between dietary intake patterns among the different geographical areas included, characterized by cultural, economic and gastronomic diversity. These differences are maintained across the different age and sex strata, coinciding with differences in the prevalence of inadequate intake of micronutrients such as vitamin A, E, B_9_, Ca and Mg. The differences found in adequate nutrient intake among the geographical areas studied could be attributable to different dietary intakes that were also influenced by sex, not being smoker, not being diabetic and being active. 

The traditional MedDiet pattern has been associated with a higher consumption of vegetables, fruits, fish, legumes and nuts [38]. However, the current dietary pattern in Spain is turning into a Westernized diet, including the overweight or obese adult population, with high consumption of sugar and meat products and a decline in the consumption of vegetables and fruits [39]. Although our results suggest that the main estimated food groups consumed were vegetables and fruits, meat and processed meat intake were elevated, while fish, seafood and legumes consumption seemed to be lower according to the recommended intakes. However, compared to a Spanish food consumption survey carried out in 2013, our estimate intake of fish and legumes was slightly higher (100.7 g/day/person versus 88.7 g/day/person and 22.1 g/day/person versus 13.9 g/day/person for fish and legumes, respectively). In contrast, egg intake was somewhat lower (23.5 versus 27.1) [40]. Egg consumption in our study was under the usually recommended levels [41], probably due to the general (but unfounded) concern that egg consumptions may increase blood cholesterol levels [42].

Depending on geographical areas, differences in food intake were found. The inhabitants of North area, especially men, consumed more sugars, sweets and alcohol than the other three areas. These data are also reflected in another Spanish study which reported that adult males from the North of Spain had higher intakes of these food groups than adult males from other Spanish areas [22]. Surprisingly, East area, the main area of fruit production in Spain, showed the lowest fruit consumption. The same was observed for olive oil consumption in South area, in spite of being the greatest olive oil producing region in the world. These data are also consistent with findings in another Spanish survey [20]. 

Food choices depend on individual’s elections, conditioned by cultural influences, demographic and socio-economic factors [43]. In addition to the geographical area, age and sex were important factors in determining food intake. According to our data, a higher consumption of vegetables, fruits and fish or seafood was found in women compared with men. Other previous studies also corroborated these findings, highlighting that females consumed more fruit and vegetables than males, decreasing the risk of nutrient deficiencies [44]. In our study, oldest people (more than 70 years) tend to have a higher consumption of vegetables, fruits, legumes, nuts and fish compared to a younger adult population. Similar findings have been previously reported in a Swiss community-dwelling sample [45]. 

People in Mediterranean countries such as Spain, have decreased their adherence to the MedDiet pattern, which was traditionally high [46]. Our results show only a moderate MedDiet adherence in most of the areas analysed. Other studies indicated a similar adherence to MedDiet at baseline interview [47]. According to the geographical area, the lowest mean score of adherence to MedDiet was found in East area, especially in younger adult men, which barely reached a compliance score of 8 out of 17 points. This finding can be explained by the low intake of fish and fruits, a surprising outcome as the geographical location of this area is on the Mediterranean coast and has an extensive agricultural tradition. This result is similar to previous findings reported in the DIMERICA study [20]. Furthermore, the percentage of the population in the first tertile of adherence to MedDiet (low adherence) reached a maximum of 48.9% in men (55–70 years) in the East area and a minimum of 16.7% in women (>70 years) in the Central area. The data described by other authors consistently show a high adherence to MedDiet in women and the aged population [48,49]. However, this phenomenon could be attributable to a generational effect (cohort effect). 

Energy and nutrient intakes in our population revealed a diet high in proteins and lipids and low in carbohydrates. In this sense, protein intake and total fat intake were above the upper recommended limits (15% and 30% of total energy intake, respectively) [50]. Other authors have shown a similar nutrient distribution in Spanish population [51]. Despite the fact that in our study total fat intake was higher than recommended for adult population, recent results from PREDIMED study have postulated that fat quality intake is a major nutritional determinant of quality of diet and cardiovascular benefits can be obtained with a relatively fat-rich diet [52]. Particularly, the adequate intake of MUFAs and PUFAs in our study, mainly due to a high consumption of olive oil, has been previously associated with a lower risk of CVD [53]. On the one hand, the intake of SFAs was not higher than 10% in most of the areas analysed, except in the East area, probably because this area recorded the highest consumption of meat and meat products compared to the other geographical territories. On the other hand, low intakes of carbohydrates and dietary fibre may be linked to low consumption of cereals, vegetables and fruits in our population study, influenced by an abandonment of traditional eating choices [54]. 

Nutrient availability can be compromised by unbalanced dietary patterns, especially for specific population groups whose requirements are increased because of age or diseases [55]. In elderly, the decrease of energy, as a result of lower food consumption has been described as an essential factor for inadequate nutrient intake [56]. Despite our population having a higher energy intake, the results in the present study suggest that they present several nutritional inadequate intakes (dietary fibre, vitamins A, B_9_, E, calcium and magnesium). Consequently, other authors put forward that micronutrient deficiencies occur frequently in overweight or obese subjects due to poor-nutritional habits and high intake of energy-dense foods [57]. In our study, the prevalence of inadequate intake was increased in elderly women but not in men for the majority of nutrients, except for calcium. This fact can be explained by an increase of milk and dairy products consumption, due to the general concern about their effects and properties on aged women bones [58]. In accordance with place of residence, the North area presented the highest prevalence of inadequate nutrient for most of the nutrients analysed, except for vitamin B_9_ and dietary fibre. This may be as consequence of their high intake of meat and meat products and cereals, as shown in Table 3, compared with the other geographical areas. 

Determinants of diet quality are multilevel and include food choices influenced by demographic variables, socioeconomic and cultural factors [59]. When comparing data from this study with a recent one which suggested that place of residence has a limited influence on dietary intake [22], our study shows that geographical area was a predictive factor of nutrient adequacy, with the highest inadequate nutrient intake in inhabitants of the North area for the majority of the nutrients analysed. In the same line, the analysis of the association between place of residence and dietary intake among North American adult women showed regional disparities in dietary intake influenced by food environment and food customs [60]. Furthermore, findings from this study, suggest that lifestyle behaviours, in particular smoking status (not being a smoker) and physical activity (being more active), were associated in a significant positive way the adequacy of nutrient intakes. Other study carried out in Brazil found a similar association for adequate minerals intake [61]. Other factors, for instance sex, have been postulated as criterion for an adequate dietary intake [62]. A study that assessed the role of diet knowledge, reported that women followed a healthy diet compared with men due to higher knowledge about food choices [63]. These results are consistent with findings in our work in which women had lower risk of inadequate nutrient intake than men. 

Finally, micronutrient intakes in elderly adults are related to socioeconomic and cultural level factors, so that participants with a high educational level and from a high social class had overall higher micronutrient intakes [64,65]. Nevertheless, we have not found this relationship, presumably because most of our participants had a similar socioeconomic and cultural level, so variability in these determinants was small.

Our study has some limitations and several strengths. Firstly, the methodological nature of a cross-sectional analysis, does not allow to address causality, although the possibility of reverse causality bias as an alternative, non-casual, explanation of our results is unlikely (nutrient intake cannot predict geographical area). Secondly, the present findings cannot be extrapolated to other population groups given that our study participants are aged adults with overweight/obesity and MetS and the sample is not representative of the general population. However, our sample is large enough to be representative of this specific population group. Thirdly, the exposure measurement of dietary intake could be influenced by the retrospective way to assess dietary intake. Although the FFQ used has been validated in adult Spanish population and has good reproducibility and a relatively good validity [28], this questionnaire may be subject to potential bias with under-reporting being a common error. For this reason, we have considered that there is an inadequate intake only when the intake does not reach 2/3 of the DRIs, with the aim to compensate the possible bias introduced by the FFQ and assuming in any case that the actual inadequate micronutrient intake should be superior to the estimated figures. However, the bias introduced would be non-differential, affecting uniformly the different geographical areas. Finally, it is unknown if the participants have always resided in the same geographical area, however, the internal migration fluxes in Spain have been low in the last twenty years, affecting mainly young people [66,67]. Among the strengths of the PREDIMED-Plus study we must point out the use of a standardized protocol which reduces information bias about food intake, socioeconomic and lifestyles variables and the election of a large sample highly representative of the Spanish older adults with MetS (*n* = 6646), as well as the inclusion of community-dwelling older population that contributes to determine the modification on nutrient intakes related to geographical disparities. Finally, to our knowledge, no study in Spain has analysed the influence of geographical area on nutrient intake in older adult population at high risk of CVD. 

## 5. Conclusions

Despite the higher figures of overweight and obesity in older adult population at high risk of CVD, there are significant inadequate nutritional intakes. Even within the same country, geographical area was significantly associated with inadequate nutrient intake. The dietary pattern of the aged adult population leaves sufficient room from improvement in relationship with the promotion of the MedDiet adherence, as well as to ensure adequate micronutrients intake. If we want to change dietetic habits in a population we need to take into account their roots and peculiarities. Therefore, it is important to show the basal differences in nutrient intake among the different study centres. This will be useful to fully understanding the results of the PREDIMED-Plus study and in addition, if this intervention is successful to extend it to other countries.

## Figures and Tables

**Table 1:** Baseline characteristics of the PREDIMED-Plus study participants by geographical areas (total population *n* = 6646).

	Total Population (*n* = 6646)	North Area (*n* = 1436)	Central Area (*n* = 493)	East Area (*n* = 2938)	South Area (*n* = 1779)	*p* Value
**Sex, *n* (%)**						
Male	3431 (51.6)	823 (57.3)	264 (53.6)	1478 (50.3)	866 (48.7)	<0.001
Female	3215 (48.4)	613 (42.7)	229 (46.5)	1460 (49.7)	913 (51.3)	
**Age, *n* (%)**						
55–70 years	5673 (85.4)	1203 (83.8)	444 (90.0)	2478 (84.3)	1548 (87.0)	<0.001
>70 years	973 (14.6)	233 (16.2)	49 (9.9)	460 (15.7)	231 (13.0)	
Mean ± SD	65.0 ± 4.9	65.2 ± 5.0	64.1 ± 4.9	65.2 ± 4.9	64.6 ± 4.8	<0.001
**Smoking habits, *n* (%)**						
Current smoker	825 (12.4)	180 (12.5)	53 (10.8)	357 (12.2)	235 (13.2)	<0.001
Former smoker	2883 (43.4)	690 (48.3)	256 (51.9)	1251 (42.6)	683 (38.4)	
Never smoker	2910 (43.8)	555 (38.7)	180 (36.5)	1325 (45.1)	850 (47.8)	
Insufficient data	28 (0.4)	8 (0.6)	4 (0.8)	5 (0.2)	11 (0.6)	
**Diabetes mellitus, *n* (%)**	1818 (27.4)	349 (24.3)	125 (25.4)	846 (28.8)	498 (28.0)	0.001
**Physical Activity, *n* (%)**						
Less active	3961 (59.8)	712 (49.8)	350 (71.4)	1720 (58.6)	1179 (66.7)	<0.001
Moderately active	1249 (18.9)	261 (18.3)	71 (14.5)	584 (19.9)	333 (18.8)	
Active	1412 (21.3)	457 (32.0)	69 (14.1)	629 (21.5)	257 (14.5)	
**Education level, *n* (%)**						
Tertiary	1463 (22.0)	305 (21.2)	181 (37.0)	654 (22.3)	323 (18.2)	<0.001
Secondary	1912 (28.8)	443 (30.9)	184 (37.6)	827 (28.2)	458 (25.7)	
Primary	3207 (48.3)	674 (46.9)	121 (24.7)	1436 (48.9)	976 (54.9)	
Insufficient data	60 (0.9)	14 (1.0)	3 (0.6)	21 (0.7)	22 (1.2)	
**Civil status, *n* (%)**						
Married	5072 (76.6)	1109 (77.9)	351 (72.1)	2240 (76.5)	1372 (77.1)	<0.001
Widowed	690 (10.4)	150 (10.5)	38 (7.8)	313 (10.7)	189 (10.2)	
Divorced/Separated	519 (7.8)	82 (5.8)	53 (10.9)	244 (8.3)	140 (7.9)	
Others ^a^	339 (5.1)	83 (5.8)	45 (9.2)	133 (4.5)	78 (4.4)	
**Living alone, *n* (%)**	826 (12.5)	180 (12.6)	77 (15.7)	365 (12.4)	204 (11.5)	0.100
**Diabetic, *n* (%)**	1818 (27.4)	349 (24.3)	125 (25.4)	846 (28.8)	498 (28.0)	0.001

Values are presented as mean ± SD for continuous variables and *n* (%) for categorical variables. Pearson’s chi-square test was performed for categorical variables and ANOVA test for continuous variables. ^a^ includes single and religious.

**Table 2:** Intake of food groups (g/day), adherence to MedDiet and nutrient profiles among geographical areas analysed (total population *n* = 6646).

	North Area		Central Area		East Area		South Area		*p* Value
*n*	(*n* = 1436)		(*n* = 493)		(*n* = 2938)		(*n* = 1779)		
	Mean	95% CI	Mean	95% CI	Mean	95% CI	Mean	95% CI	
**Food group intake**									
Vegetables (g/day)	291.1	284.1–298.2	327.8	315.1–340.5	335.8	330.8–34.7	331.5	325.1–337.9	<0.001
Fruits (g/day)	389.6	380.0–400.1	366.0	347.0–385.0	332.5	325.1–339.8	366.9	357.4–376.5	0.001
Legumes (g/day)	18.8	18.2–19.4	20.6	19.6–21.7	19.7	19.3–20.1	23.2	22.7–23.8	<0.001
Cereals (g/day)	173.4	169.4–177.4	150.2	143.0–157.3	142.8	140.0–145.5	143.8	140.2–147.4	<0.001
Milk/dairy products (g/day)	367.0	356.6–377.3	387.3	368.7–405.9	320.8	313.6–328.1	357.9	348.6–367.3	<0.001
Meat/meat products (g/day)	150.8	147.9–153.7	143.6	138.4–148.8	155.4	153.4–157.5	125.7	123.1–128.3	<0.001
Olive oil (g/day)	42.9	42.0–43.8	35.9	34.4–37.5	40.6	40.0–41.2	37.0	36.2–37.8	<0.001
Fish/seafood (g/day)	95.7	93.3–98.1	109.8	105.4–114.1	102.5	100.9–104.2	99.5	97.3–101.7	<0.001
Nuts (g/day)	12.9	12.–13.8	16.3	14.7–17.9	15.0	14.4–15.6	15.5	14.7–16.3	<0.001
Sugar/sweets (g/day)	32.6	31.1–34.2	29.5	26.7–32.2	25.5	24.5–26.6	23.2	21.8–24.6	<0.001
Eggs (g/day)	25.9	25.3–26.5	24.1	23.0–25.2	22.6	22.1–23.0	23.0	22.5–23.6	<0.001
**Adherence to MedDiet**									
MedDiet Q-P17 ^a^, mean	8.6	8.4–8.7	9.2	9.0–9.5	8.1	8.0–8.2	8.9	8.8–9.0	<0.001
**Energy intake and nutrient profiles, mean**									
Total energy intake (kcal/day)	2425.0	2397.6–2452.3	2398.8	2349.7–2447.9	2357.6	2338.5–2376.6	2301.0	2276.3–2325.7	<0.001
Total fat intake (%)	38.3	38.0–38.6	38.5	37.9–39.1	40.4	40.1–40.6	39.0	38.7–39.3	<0.001
Monounsaturated fat (%)	19.9	19.7–20.2	19.5	19.1–19.9	21.0	20.8–21.1	20.3	20.1–20.5	<0.001
Polyunsaturated fat (%)	6.1	6.0–6.2	6.3	6.1–6.5	6.4	6.3–6.4	6.5	6.4–6.6	<0.001
Saturate fat (%)	9.5	9.4–9.6	10.1	9.9–10.2	10.3	10.2–10.3	9.6	9.5–9.6	<0.001
Carbohydrate intake (%)	41.8	41.1–42.1	41.6	41.0–42.3	39.8	39.6–40.1	41.8	41.5–42.1	<0.001
Protein intake (%)	16.3	16.1–16.4	17.0	16.7–17.2	16.8	16.7–16.9	16.4	16.3–16.5	<0.001
Alcohol intake (g/day)	13.5	12.8–14.2	10.4	9.1–11.6	10.6	10.1–11.1	9.7	9.1–10.4	<0.001
Fibre intake (g/day)	25.8	25.4–26.3	26.4	25.6–27.3	25.8	25.4–26.1	26.2	25.8–26.6	0.191

Values are presented as means adjusted by age and sex. ANCOVA test was performed. ^a^ MedDiet Q-P17, adherence to Mediterranean diet questionnaire 17 point cut off.

**Table 3:** Participants with nutrient intake below 2/3 of DRIs by geographical areas, age and sex.

Nutrient	Group	DRI ^a^	North Area	Central Area	East Area	South Area	*p* Value ^1^
**Dietary fibre**	Male 55–70	30 g/day	28.1	28.4	27.0	29.2	0.744
	Male >70	30 g/day	19.4	27.8	24.0	29.4	0.442
	Female 60–70	21 g/day	2.3	4.6	4.1	4.0	0.310
	Female >70	21 g/day	3.7	6.9	5.6	2.2	0.383
***p* value ^2^**			<0.001	<0.001	<0.001	<0.001	
**Vitamin A**	Male 55–70	900 µg/day	28.8	21.2	15.8	16.6	<0.001
	Male >70	900 µg/day	25.5	16.7	17.3	17.4	0.353
	Female 60–70	700 µg/day	8.6	5.8	5.6	5.2	0.078
	Female >70	700 µg/day	8.2	6.9	10.4	5.1	0.341
***p* value *^2^***			<0.001	<0.001	<0.001	<0.001	
**Vitamin B_9_**	Male 60–70	400 µg/day	19.3	18.5	22.2	22.6	0.250
	Male >70	400 µg/day	17.4	22.2	18.8	23.9	0.667
	Female 55–70	400 µg/day	15.7	17.3	17.4	20.2	0.204
	Female >70	400 µg/day	11.9	24.1	21.9	21.0	0.092
***p* value ^2^**			0.128	0.819	0.020	0.649	
**Vitamin D**	Male 60–70	15 µg/day	89.0	75.7	83.6	83.0	<0.001
	Male >70	20 µg/day	99.0	100.0	97.6	97.8	0.786
	Female 55–70	15 µg/day	87.9	77.5	82.0	85.0	0.002
	Female >70	20 µg/day	98.5	93.1	97.2	100.0	0.061
***p* value ^2^**			<0.001	0.021	<0.001	<0.001	
**Vitamin E**	Male 55–70	15 mg/day	63.7	52.3	43.3	43.6	<0.001
	Male >70	15 mg/day	56.1	50.0	44.7	54.4	0.214
	Female 60–70	15 mg/day	63.1	48.6	49.0	50.4	<0.001
	Female >70	15 mg/day	70.2	44.8	61.8	61.6	0.062
***p* value ^2^**			0.178	0.826	<0.001	<0.001	
**Calcium**	Male 55–70	1000 mg/day	14.2	8.6	11.7	13.5	0.090
	Male >70	1200 mg/day	22.5	11.1	30.8	30.4	0.163
	Female 60–70	1200 mg/day	25.3	19.7	26.7	25.7	0.268
	Female >70	1200 mg/day	21.6	27.6	24.3	28.3	0.625
***p* value ^2^**			<0.001	0.002	<0.001	<0.001	
**Magnesium**	Male 55–70	420 mg/day	10.2	8.1	7.6	7.7	0.206
	Male >70	420 mg/day	12.2	5.6	8.7	9.8	0.718
	Female 60–70	320 mg/day	1.0	1.7	1.2	1.5	0.846
	Female >70	320 mg/day	1.5	3.5	1.2	1.5	0.819
***p* value ^2^**			<0.001	0.042	<0.001	<0.001	

DRI ^a^: Dietary Reference Intake. Pearson’s Chi-Square test was used to estimate differences among prevalence of inadequate nutrient intakes according to geographical area for each age and sex strata (*p* value ^1^) and also to estimate differences among prevalence of inadequate nutrient intakes according to age and sex, for each geographical area (*p* value ^2^).

**Table 4:** Results from the logistic regression model of micronutrient inadequate intakes according to 2/3 DRIs by geographical areas.

Nutrient		North Area	Central Area	East Area	South Area
Dietary fibre	Model 1	1 (Ref.)	1.08 (0.81−1.43)	0.92 (0.78−1.10)	0.97 (0.80−1.17)
	Model 2	1 (Ref.)	1.03 (0.72−1.46)	0.80 (0.65−0.98)	0.92 (0.73−1.15)
Vitamin A	Model 1	1 (Ref.)	0.66 (0.49−0.89)	0.51 (0.43−0.61)	0.49 (0.40−0.59)
	Model 2	1 (Ref.)	0.57 (0.41−0.80)	0.43 (0.35−0.52)	0.40 (0.32−0.50)
Vitamin B_9_	Model 1	1 (Ref.)	1.09 (0.83−1.44)	1.19 (1.01−1.41)	1.31 (1.10−1.57)
	Model 2	1 (Ref.)	0.98 (0.70−1.36)	0.97 (0.80−1.17)	1.09 (0.88−1.34)
Vitamin E	Model 1	1 (Ref.)	0.58 (0.47−0.72)	0.52 (0.45−0.59)	0.54 (0.47−0.62)
	Model 2	1 (Ref.)	0.47 (0.36−0.61)	0.30 (0.26−0.35)	0.30 (0.25−0.36)
Calcium	Model 1	1 (Ref.)	0.70 (0.52−0.94)	1.07 (0.91−1.26)	1.11 (0.93−1.32)
	Model 2	1 (Ref.)	0.53 (0.38−0.76)	0.86 (0.72−1.04)	0.80 (0.65−0.98)
Magnesium	Model 1	1 (Ref.)	0.80 (0.50−1.28)	0.68 (0.52−0.89)	0.70 (0.51−0.95)
	Model 2	1 (Ref.)	0.39 (0.21−0.75)	0.35 (0.25−0.50)	0.33 (0.22−0.49)

Values are presented as OR and 95% CI for the inadequate intake of micronutrients as categorical variable according to area of residence. Model 1: This model has not been adjusted for any variable. Model 2: has been adjusted by sex, age, smoking habits, physical activity, educational status, diabetic status, living alone, total energy intake and adherence to MedDiet.

**Table 5:** Multivariable logistic regression model for inadequate intake of 3 or more out 6 micronutrients according to geographical area.

	**≥3 Inadequate Intake % Prevalence (95% CI)**	**Adjusted Odds Ratio (95% CI)**	***p*** **Value**
**Geographical area**			
North area	19.0 (17.0–21.1)	1 (Ref.)	
Central area	16.3 (12.8–19.7)	0.65 (0.46–0.94)	0.021
East area	15.9 (14.6–17.2)	0.57 (0.47–0.70)	<0.001
South area	16.8 (15.0–18.5)	0.59 (0.47–0.74)	<0.001

## Supplementary Materials

The following materials are available online at http://www.mdpi.com/2072-6643/10/11/1661/s1, Table S1: Intake of food groups (g/day) in both sexes divided by geographical areas and age, Table S2: MedDiet adherence, total energy intake and profile of macronutrients, fibre and alcohol intake by geographical areas (total population *n* = 6646) among 55–75 years population, Table S3: Participants with nutrient intake below AR/AI proposed by EFSA by geographical areas, age and sex, Table S4: Logistic regression model of micronutrients inadequate intake according to EFSA by geographical areas, Table S5: Logistic regression model of micronutrients deficiency intake according to 2/3 DRIs by areas, Table S6: Logistic regression model of micronutrients deficiency intake according to EFSA by areas, Table S7: Multivariable logistic regression model for inadequate intake of 3 or more out 6 micronutrients in accordance with criteria by EFSA according to geographical area.
