# Supplementary material for: Dietary Intake in Population with Metabolic Syndrome: Is the Prevalence of Inadequate Intake Influenced by Geographical Area? Cross-Sectional Analysis from PREDIMED-Plus Study
Source: Nutrients. 2018 Nov 4;10(11):1661. doi: 10.3390/nu10111661 (PMC6266979; doi:10.3390/nu10111661)
Supplement: Supplementary file 1 [file nutrients-10-01661-s001.pdf]

nutrients

MDPI

Article

Dietary Intake in Population with Metabolic Syndrome: Is the Prevalence of Inadequate Intake Influenced by Geographical Area? Cross-Sectional Analysis from PREDIMED-Plus Study

Naomi Cano-Ibáñez [1,2,*], Aurora Bueno-Cavanillas [1,2], Miguel A. Martínez-González [3,4,5], Dolores Corella [5,6], Jordi Salas-Salvadó [5,7], M. Dolors Zomeño [8,9], Manoli García-de-la-Hera [2,10], Dora Romaguera [5,11], J. Alfredo Martínez [5,12,13], F. Javier Barón-López [5,14], Antonio García-Ríos [5,15], Ramón Estruch [5,16], Laura García-Molina [1,2], Ángel Alonso Gómez [5,17], Josep A. Tur [5,18], Francisco Tinahones [5,19], Lluis Serra-Majem [5,20], Naiara Cubelos-Fernández [21], José Lapetra [5,22], Clotilde Vázquez [5,23], Xavier Pintó [5,24], Josep Vidal [25,26], Lidia Daimiel [13], José Juan Gaforio [2,27], Pilar Matía [28], Emilio Ros [5,29], Javier Diez-Espino [3,5,30], Rebeca Fernández-Carrión [5,6], Josep Basora [5,7], Montse Fitó [5,8], Juan Manuel Zazo [2,10], Antoni Colom [5,11], Estefanía Toledo [3,5], Andrés Díaz-López [5,7], Miguel Ángel Muñoz [31], Miguel Ruiz-Canela [3,4,5] and Alfredo Gea [3,5]

Supplementary Materials:

Table S1. Intake of food groups (g/day) in both sexes divided by geographical areas and age.

	55-70 years									
	**Men**					**Women**				
	**North area (n=724)**	**Central area (n=245)**	**East area (n=1269)**	**South area (n=774)**	***P value***	**North area (n=479)**	**Central area (n=199)**	**East area (n=1209)**	**South area (n=774)**	***P value***
Vegetables	277.1 ±95.3	304.4 ±155.8	317.5 ±141.9	314.1±141.4	<0.001	301.2 ±106.8	357.2 ±163.9	358.6 ±144.7	352.3 ±146.0	<0.001
Fruits	354.8±204.8	346.5±259.1	315.5±184.3	339.5±195.9	0.001	412.2±225.2	369.1±242.6	343.2±187.4	387.5±215.6	<0.001
Legumes	18.9±8.2	20.6±10.9	20.2±11.5	23.0±10.6	<0.001	17.7±7.8	20.8±13.9	19.2±10.9	23.7±11.5	<0.001
Cereals	190.5±100.0	156.9±92.3	153.4±76.5	152.1±75.9	<0.001	152.8±77.3	142.6±87.4	132.5±66.7	135.0±63.7	<0.001
Milk/dairy products	333.9±206.3	379.3±220.1	306.4±188.7	337.1±200.1	<0.001	401.0±208.4	377.9±177.9	334.6±193.1	375.7±214.8	<0.001
Meat/meat products	155.7±54.8	153.4±58.7	163.0±63.0	137.7±57.4	<0.001	147.7±51.0	134.8±51.3	150.1±56.1	117.7±48.5	<0.001
Olive oil	43.2±13.5	35.4±20.5	41.1±16.7	39.6±19.0	<0.001	43.3±14.7	36.8±18.6	39.9±16.7	33.4±17.5	<0.001
Fish/seafood	92.9±40.6	107.8±53.6	99.0±48.1	102.3±48.9	<0.001	99.8±38.1	114.2±67.5	106.3±45.8	99.0±46.1	<0.001
Nuts	13.2±16.1	16.3±20.9	15.5±17.3	15.3±16.5	0.018	12.0±16.5	15.5±23.1	15.0±17.3	16.0±18.2	0.001
Sugar/sweets	34.3±37.6	31.8±30.6	27.8±30.2	23.3±24.1	<0.001	31.0±34.1	27.7±31.0	23.5±24.9	22.8±24.9	<0.001
Eggs	27.7±14.8	24.9±14.1	23.7±12.4	23.7±12.8	<0.001	24.1±11.4	23.2±14.7	21.8±9.2	22.4±10.8	0.006

	**More than 70 years**									
	**Men**					**Women**				
Vegetables	298.6±113.7	308.6±120.0	325.0±143.7	326.9±128.3	0.368	304.9±105.7	333.3±137.6	332.1±141.7	330.0±136.2	0.245
Fruits	403.6±191.9	330.4±219.9	353.0±195.8	342.8±164.6	0.087	466.5±194.9	481.3±383.6	359.4±198.9	429.2±235.1	<0.001
Legumes	21.1±8.6	25.8±18.1	21.0±12.9	22.7±10.5	0.280	20.8±15.3	17.1±11.9	18.3±9.4	22.1±11.5	0.008
Cereals	187.0±95.9	152.7±82.7	154.6±76.4	147.6±82.7	0.004	158.8±88.0	153.0±98.1	124.8±58.5	135.7±69.1	0.001
Milk/dairy products	321.7±185.4	409.3±234.8	295.5±172.4	334.2±172.3	0.035	432.7±197.5	465.5±221.1	356.3±196.8	403.6±220.2	0.001
Meat/meat products	149.2±55.3	151.4±46.6	153.5±55.2	109.7±41.6	<0.001	143.3±50.6	127.2±45.5	141.5±53.0	111.9±47.2	<0.001
Olive oil	42.6±13.8	36.5±21.0	40.7±16.6	35.1±17.5	0.064	41.0±14.1	35.7±17.5	41.2±15.6	33.4±17.5	<0.001
Fish/seafood	94.6±41.5	105.2±52.8	102.3±45.0	94.2±47.3	0.327	94.2±38.5	101.6±62.6	102.9±48.4	92.4±40.3	0.104
Nuts	14.6±17.9	17.6±19.2	15.5±16.2	14.8±15.3	0.891	13.2±14.5	21.6±27.7	12.0±13.5	14.2±14.8	0.001
Sugar/sweets	35.2±39.1	29.9±57.5	26.7±31.5	28.6±43.7	0.324	29.9±32.0	23.6±20.5	22.0±24.5	20.3±22.5	0.012
Eggs	25.8±16.7	25.3±14.6	21.6±9.6	23.4±10.4	0.036	23.9±9.9	24.4±10.3	21.3±9.6	22.3±9.2	0.056

Data are expressed as means ± SD. ANOVA test was performed.

Table S2. MedDiet adherence, total energy intake and profile of macronutrients, fiber and alcohol intake by geographical areas (total population *n = 6646*) among 55-75 years population.

	55-70 years									
	**Men**					**Women**				
	**North area (n=724)**	**Central area (n=245)**	**East area (n=1269)**	**South area (n=774)**	***P value***	**North area (n=479)**	**Central area (n=199)**	**East area (n=1209)**	**South area (n=774)**	***P value***
MedDiet adherence Mean ±SD	8.1±2.7	9.0±2.4	7.7±2.6	8.5±2.6	<0.001	9.0±2.7	9.4±2.4	8.5±2.6	9.2±2.5	<0.001
Low adherence, n (%)	307 (42.4)	77 (31.4)	621 (48.9)	269 (34.8)		146 (30.5)	43 (21.6)	435 (35.6)	186 (24.0)	
Medium adherence, n (%)	272 (37.6)	106 (43.3)	466 (36.7)	330 (42.7)	<0.001	188 (39.3)	89 (44.7)	502 (41.5)	356 (46.09	<0.001
High adherence, n (%)	145 (20.0)	62 (25.3)	182 (14.3)	175 (22.6)		145 (30.3)	67 (33.7)	272 (22.5)	232 (30.0)	
Total energy intake, mean ±sd	2575±533	2507±629	2524±5446	2456±572	0.001	2262±501	2293±519	2205±485	2173±501	0.002
%Total fat intake,mean ±sd	37.6±5.9	38.0±6.5	40.0±6.0	39.5±6.9	<0.001	39.4±6.3	39.1±7.1	40.8±6.4	38.9±7.1	<0.001
% Monounsaturated fat,mean ±sd	19.4±4.0	19.0±4.5	20.8±4.3	20.6±5.1	<0.001	20.8±4.3	20.1±5.3	21.3±4.7	20.3±5.1	<0.001
% Polyunsaturated fat,mean ±sd	6.0±1.9	6.3±2.0	6.3±1.7	6.5±1.6	<0.001	6.1±2.0	6.3±1.9	6.5±1.9	6.5±1.8	0.002
% Saturated fat, mean ±sd	9.4±1.8	10.1±2.1	10.3±2.0	9.7±1.8	<0.001	9.7±2.0	10.1±2.2	10.3±2.0	9.5±1.9	<0.001
% Carbohydrate intake,mean ±sd	41.2±6.6	41.0±6.3	39.5±6.5	40.4±7.1	<0.001	41.9±6.4	42.2±7.2	40.1±6.6	42.9±7.2	<0.001
Protein intake (%),mean ±sd	15.5±2.4	16.6±3.2	16.0±2.7	15.8±2.7	<0.001	17.2±2.5	17.3±3.0	17.7±2.8	17.0±2.6	<0.001
Alcohol intake (g/d),mean ±sd	21.3±20.9	16.3±17.6	16.2±16.4	15.5±15.7	<0.001	5.1±10.2	4.7±7.6	4.5±6.9	4.1±6.9	0.162
Fiber intake (g/d),mean ±sd	25.1±8.6	25.3±9.2	25.2±8.5	24.9±8.6	0.883	25.9±8.3	27.2±9.7	26.3±8.5	27.6±9.4	0.003
	**More than 70 years**									
	**Men**					**Women**				
MedDiet Q-P17[a] adherence Mean ±SD	8.5±3.1	8.5±2.4	8.3±2.6	9.0±2.5	0.275	9.3±2.6	9.5±2.0	8.5±21	9.4±2.5	0.002
Low adherence, n (%)	38 (38.4)	5 (26.3)	80 (30.3)	30 (32.6)		32 (23.9)	5 (16.7)	89 (35.5)	33 (23.7)	
Medium adherence, n (%)	33 (33.3)	10 (52.6)	89 (42.6)	41 (44.6)	0.622	59 (44.0)	16 (53.3)	103 (41.0)	54 (38.9)	0.004

High adherence, n (%)	28 (28.3)	4 (21.1)	40 (19.1)	21 (22.8)		43 (32.1)	9 (30.0)	59 (23.5)	52 (37.4)	
Total energy intake, mean ±sd	2574.5±602.3	2439.1±534.9	2444.4±527.7	2257.4±548.4	0.001	2264.2±527.9	2338.7±575.1	2127.1±461.6	2085.3±484.5	0.003
%Total fat intake,mean ±sd	37.7±6.1	38.3±8.3	39.5±6.4	38.9±7.0	0.154	38.0±6.7	38.9±7.7	40.5±6.0	37.7±7.3	0.001
% Monounsaturate fat, mean ±sd	19.3±3.5	19.4±4.9	20.5±4.6	20.1±5.2	0.182	19.7±4.4	20.2±5.8	21.1±4.2	19.5±5.1	0.002
% Polyunsaturate fat, mean ±sd	6.3±2.3	6.4±2.5	6.4±1.8	6.7±2.0	0.671	6.2±1.9	6.7±2.2	6.3±1.7	6.4±1.9	0.429
% Saturated fat,mean ±sd	9.4±1.8	10.0±2.5	10.1±2.0	9.5±1.7	0.008	9.3±1.9	9.6±2.3	10.2±2.0	9.1±1.9	<0.001
% Carbohydrate intake,mean ±sd	41.6±6.8	40.5±8.2	39.9±6.4	41.4±7.1	0.148	43.6±7.0	43.0±8.2	40.5±6.4	44.3±7.1	<0.001
Protein intake (%),mean ±sd	15.5±2.0	17.2±3.2	16.1±2.4	15.7±2.6	0.015	17.3±2.3	16.9±2.6	17.6±2.9	17.1±2.6	0.255
Alcohol intake (g/d),mean ±sd	20.2±18.7	12.2±12.7	15.9±15.9	12.9±12.6	0.010	3.5±6.7	3.6±3.8	4.5±7.6	2.7±5.0	0.064
Fiber intake (g/d),mean ±sd	26.9±8.1	27.5±9.8	26.7±9.2	25.3±8.5	0.511	28.2±9.0	29.3±11.5	25.5±8.0	27.1±8.5	0.010

Values are means ± SD for continuous variables and percentages (n) for categorical variables unless otherwise indicated. Abbreviations: (MedDietQ-P17[b]), Mediterranean diet questionnaire 17 point cut off

Table S3. Participants with nutrient intake below AR/AI proposed by EFSA by geographical areas, age and sex.

Nutrient	*Group*	*AR/AI*[a]	*North area*	*Central area*	*East area*	*South area*	*P value*[1]
**Dietary fiber**	Male	*25g /d*	55.3	53.3	54.2	58.9	0.183
	Female	*25g /d*	49.8	48.5	48.7	45.2	0.273
**P *value*[2]**			0.037	0.313	0.003	<0.001	
**Vitamin A**	Male	*570 µg/d*	23.5	19.2	14.2	14.5	<0.001
	Female	*490 µg/d*	9.8	7.4	7.8	6.6	0.154
**P *value*[2]**			<0.001	<0.001	<0.001	<0.001	
**Vitamin B_9**	Male	*250 µg/d*	13.6	15.4	16.5	17.1	0.201
	Female	*250 µg/d*	11.3	13.4	14.5	14.8	0.196
**P *value*[2]**			0.189	0.542	0.123	0.202	
**Vitamin D**	Male	*15 µg/d*	99.3	95.0	99.1	99.1	<0.001
	Female	*15 µg/d*	99.5	96.5	99.0	99.1	0.004
**P *value*[2]**			0.562	0.428	0.680	0.914	
**Vitamin E**	Male	*13 mg/d*	86.4	77.5	75.5	75.7	<0.001
	Female	*11 mg/d*	76.0	60.4	65.0	63.2	<0.001
**P *value*[2]**			<0.001	<0.001	<0.001	<0.001	
**Calcium**	Male	*950 mg/d*	48.6	37.5	46.5	45.8	0.026
	Female	*950 mg/d*	43.4	37.1	46.7	43.5	0.045
**P *value*[2]**			0.051	0.936	0.871	0.336	
**Magnesium**	Male	*350 mg/d*	34.9	29.2	31.3	32.4	0.233
	Female	*350 mg/d*	18.8	12.9	16.2	16.3	0.232
**P *value*[2]**			<0.001	<0.001	<0.001	<0.001	

[a]AR/AI: Average Requirements/Adequate intake according to EFSA criteria. Pearson`s Chi Square test was used to estimate differences among prevalence of inadequate nutrient intakes according to geographical area for sex strata (*p value*[1]) and also to estimate differences among prevalence of inadequate nutrient intakes according to sex, for each geographical area (*p value*[2]).

Table S4. Logistic regression model of micronutrients inadequate intake according to EFSA by geographical areas.

Nutrient		*North area*	*Central area*	*East area*	*South area*
**Dietary fiber**	Model 1	1 (Ref.)	0.93 (0.75-1.15)	0.94 (0.83-1.08)	0.95 (0.83-1.10)
	Model 2	1 (Ref.)	0.92 (0.71-1.19)	0.72 (0.62-0.84)	0.78 (0.66-0.91)
**Vitamin A**	Model 1	1 (Ref.)	0.75 (0.55-1.01)	0.58 (0.48-0.69)	0.54 (0.44-0.67)
	Model 2	1 (Ref.)	0.68 (0.49-0.96)	0.49 (0.40-0.59)	0.45 (0.36-0.56)
**Vitamin B9**	Model 1	1 (Ref.)	1.18 (0.86-1.60)	1.27 (1.06-1.53)	1.32 (1.08-1.61)
	Model 2	1 (Ref.)	1.05 (0.73-1.51)	1.02 (0.83-1.26)	1.06 (0.84-1.34)
**Vitamin E**	Model 1	1 (Ref.)	0.51 (0.40-0.65)	0.52 (0.44-0.61)	0.50 (0.42-0.59)
	Model 2	1 (Ref.)	0.45 (0.34-0.61)	0.36 (0.30-0.43)	0.33 (0.27-0.40)
**Calcium**	Model 1	1 (Ref.)	0.69 (0.55-0.86)	1.01 (0.89-1.15)	0.93 (0.81-1.07)
	Model 2	1 (Ref.)	0.56 (0.43-0.73)	0.87 (0.75-1.00)	0.70 (0.59-0.82)
**Magnesium**	Model 1	1 (Ref.)	0.71 (0.55-0.92)	0.80 (0.70-0.93)	0.82 (0.70-0.96)
	Model 2	1 (Ref.)	0.55 (0.38-0.80)	0.47 (0.39-0.58)	0.45 (0.36-0.56)

Values are presented as OR and 95% CI for the inadequacy intake of micronutrients as categorical variable according to area of residence. Model 1: This model has not been adjusted for any variable. Model 2: has been adjusted by sex, age, smoking habits, physical activity, educational status, diabetic status, living alone, total energy intake and adherence to MedDiet.

Table S5. Logistic regression model of micronutrients deficiency intake according to 2/3 DRIs by areas.

	DIETARY FIBER		*VITAMIN A*		*VITAMIN B_9*		*VITAMIN E*		*CALCIUM*		*MAGNESIUM*	
	**OR (CI 95%)**	***p value***	**OR (CI 95%)**	***p value***	**OR (CI 95%)**	***p value***	**OR (CI 95%)**	***p value***	**OR (CO 95%)**	***p value***	**OR (CI 95%)**	***p value***
**Geographical areas**												
North area	1 (Ref.)		1 (Ref.)		1 (Ref.)		1 (Ref.)		1 (Ref.)		1 (Ref.)	
Central area	1.03 (0.72-1.46)	0.874	0.57 (0.41-0.80)	0.001	0.98 (0.70-1.36)	0.889	0.47 (0.36-0.61)	<0.001	0.53 (0.38-0.76)	<0.001	0.39 (0.21-0.75)	0.005
East area	0.80 (0.65-0.98)	0.033	0.43 (0.35-0.52)	<0.001	0.97 (0.80-1.17)	0.721	0.30 (0.26-0.35)	<0.001	0.86 (0.72-1.04)	0.112	0.35 (0.25-0.50)	<0.001
South area	0.92	0.454	0.40	<0.001	1.09	0.437	0.30	<0.001	0.80	0.029	0.33	<0.001

	(0.73-1.15)		(0.32-0.50)		(0.88-1.34)		(0.25-0.36)		(0.65-0.98)		(0.22-0.49)	
**Age**	0.83 (0.67-1.06)	0.133	1.04 (0.84-1.30)	0.706	0.93 (0.77-1.14)	0.507	1.25 (1.06-1.48)	0.010	1.41 (1.17-1.70)	<0.001	0.92 (0.62-1.38)	0.713
**Sex**												
Men	1 (Ref.)		1 (Ref.)		1 (Ref.)		1 (Ref.)		1 (Ref.)		1 (Ref.)	
Female	0.05 (0.04-0.07)	<0.001	0.19 (0.16-0.23)	<0.001	0.49 (0.42-0.58)	<0.001	0.66 (0.58-0.76)	<0.001	1.36 (1.15-1.60)	<0.001	0.93 (0.62-1.38)	0.713
**MedDiet adherence**												
Low adherence	1 (Ref.)		1 (Ref.)		1 (Ref.)		1 (Ref.)		1 (Ref.)		1 (Ref.)	
Moderate adherence	0.41 (0.35-0.49)	<0.001	0.71 (0.60-0.84)	<0.001	0.47 (0.40-0.54)	<0.001	0.58 (0.51-0.67)	<0.001	0.78 (0.66-0.91)	0.002	0.25 (0.18-0.35)	<0.001
High adherence	0.14 (0.11-0.18)	<0.001	0.44 (0.35-0.55)	<0.001	0.19 (0.16-0.24)	<0.001	0.35 (0.30-0.41)	<0.001	0.58 (0.48-0.71)	<0.001	0.09 (0.06-0.15)	<0.001
**Energy intake (kcal/day)**	0.99 (0.99-0.99)	<0.001	0.99 (0.99-0.99)	<0.001	0.99 (0.99-0.99)	<0.001	0.99 (0.99-0.99)	<0.001	0.99 (0.99-0.99)	<0.001	0.99 (0.99-0.99)	<0.001
**Smoking habits**												
Current smoker	1 (Ref.)		1 (Ref.)		1 (Ref.)		1 (Ref.)		1 (Ref.)		1 (Ref.)	
Former smoker	0.71 (0.57-0.88)	0.002	1.09 (0.86-1.38)	0.472	0.69 (0.56-0.86)	0.001	0.84 (0.69-1.01)	0.119	0.84 (0.67-1.06)	0.125	0.60 (0.41-0.87)	0.007
Never smoker	0.67 (0.52-0.85)	0.001	0.97 (0.75-1.25)	0.798	0.60 (0.48-0.75)	<0.001	0.82 (0.67-1.00)	0.047	0.68 (0.54-0.86)	0.001	0.51 (0.33-0.79)	0.003
**Physical activity**												
Less active	1 (Ref.)		1 (Ref.)		1 (Ref.)		1 (Ref.)		1 (Ref.)		1 (Ref.)	
Moderate active	0.58 (0.47-0.72)	<0.001	0.82 (0.67-1.01)	0.064	0.65 (0.53-0.79)	<0.001	0.96 (0.82-1.12)	0.578	0.83 (0.69-1.00)	0.056	0.52 (0.35-0.77)	0.001
Active	0.83 (0.67-1.01)	0.065	0.88 (0.72-1.07)	0.187	0.88 (0.74-1.06)	0.187	0.93 (0.79-1.08)	0.323	1.00 (0.84-1.20)	0.982	0.83 (0.58-1.19)	0.306
**Education level**												
Tertiary level	1 (Ref.)		1 (Ref.)		1 (Ref.)		1 (Ref.)		1 (Ref.)		1 (Ref.)	
Secondary level	0.91 (0.74-1.12)	0.376	0.81 (0.66-1.01)	0.048	1.02 (0.84-1.24)	0.829	1.20 (1.01-1.42)	0.035	1.02 (0.83-1.25)	0.869	1.00 (0.68-1.46)	0.984
Primary level	0.75 (0.62-0.92)	0.006	0.88 (0.73-1.08)	0.221	0.80 (0.67-0.97)	0.022	1.12 (0.95-1.31)	0.177	1.04 (0.85-1.25)	0.725	1.22 (0.85-1.76)	0.277
**Non diabetic**	0.79 (0.66-0.94)	0.008	0.81 (0.68-0.97)	0.019	0.95 (0.80-1.10)	0.481	0.85 (0.75-0.97)	0.019	0.79 (0.68-0.93)	0.004	0.81 (0.59-1.10)	0.176
**Living alone**	0.84 (0.64-1.10)	0.193	0.91 (0.71-1.17)	0.466	0.82 (0.67-1.01)	0.068	0.93 (0.77-1.11)	0.426	1.09 (0.89-1.35)	0.376	1.31 (0.80-2.13)	0.285

OR, p value and 95% CI for the inadequacy intake of micronutrients as categorical variable according to area. Model has been adjusted by sex, age, smoking habits, physical activity, educational status, diabetic status, living alone, total energy intake and adherence into MedDiet Q-P17.

Table S6. Logistic regression model of micronutrients deficiency intake according to EFSA by areas.

	DIETARY FIBER		*VITAMIN A*		*VITAMIN B_9*		*VITAMIN E*		*CALCIUM*		*MAGNESIUM*	
	**OR (CI 95%)**	**p value**	**OR (CI 95%)**	**p value**	**OR (CI 95%)**	**p value**	**OR (CI 95%)**	**p value**	**OR (CO 95%)**	**p value**	**OR (CI 95%)**	**p value**
**Geographical areas**												
North area	1 (Ref.)		1 (Ref.)		1 (Ref.)		1 (Ref.)		1 (Ref.)		1 (Ref.)	
Central area	0.92 (0.71-1.19)	0.518	0.68 (0.49-0.96)	0.026	1.05 (0.73-1.50)	0.798	0.48 (0.34-0.61)	<0.001	0.56 (0.44-0.73)	<0.001	0.51 (0.36-0.74)	>0.001
East area	0.72 (0.62-0.84)	<0.001	0.49 (0.40-0.59)	<0.001	1.02 (0.83-1.26)	0.859	0.36 (0.30-0.43)	<0.001	0.87 (0.75-1.00)	0.051	0.48 (0.39-0.59)	<0.001
South area	0.78 (0.66-0.92)	0.003	0.45 (0.36-0.56)	<0.001	1.06 (0.84-1.34)	0.615	0.33 (0.27-0.40)	<0.001	0.70 (0.59-0.82)	<0.001	0.43 (0.35-0.54)	<0.001
**Age**	0.79 (0.67-0.93)	0.005	0.11 (0.76-1.20)	0.703	0.92 (0.73-1.14)	0.432	1.16 (0.96-1.39)	0.125	0.95 (0.81-1.12)	0.544	0.96 (0.77-1.20)	0.715
**Sex**												
Men	1 (Ref.)		1 (Ref.)		1 (Ref.)		1 (Ref.)		1 (Ref.)		1 (Ref.)	
Female	0.47 (0.41-0.54)	<0.001	0.27 (0.23-0.33)	<0.001	0.53 (0.44-0.63)	<0.001	0.21 (0.18-0.25)	<0.001	0.52 (0.46-0.60)	<0.001	0.08 (0.07-0.10)	<0.001
**MedDiet adherence**												
Low adherence	(Ref.)		(Ref.)		1 (Ref.)		1 (Ref.)		1 (Ref.)		1 (Ref.)	
Moderate adherence	0.37 (0.33-0.43)	<0.001	0.68 (0.57-0.81)	<0.001	0.46 (0.39-0.55)	<0.001	0.66 (0.56-0.76)	<0.001	0.90 (0.79-1.02)	0.108	0.35 (0.29-0.42)	<0.001
High adherence	0.14 (0.12-0.17)	<0.001	0.40 (0.31-0.50)	<0.001	0.18 (0.14-0.23)	<0.001	0.43 (0.36-0.51)	<0.001	0.68 (0.59-0.80)	<0.001	0.10 (0.08-0.13)	<0.001
**Energy intake (kcal/day)**	0.99 (0.99-0.99)	<0.001	0.99 (0.99-0.99)	<0.001	0.99 (0.99-0.99)	<0.001	0.99 (0.99-0.99)	<0.001	0.99 (0.99-0.99)	<0.001	0.99 (0.99-0.99)	<0.001
**Smoking habits**												
Current smoker	1 (Ref.)		1 (Ref.)		1 (Ref.)		1 (Ref.)		1 (Ref.)		1 (Ref.)	
Former smoker	0.70 (0.58-0.85)	<0.001	1.16 (0.91-1.48)	0.241	0.67 (0.53-0.85)	0.001	0.87 (0.70-1.08)	0.204	0.81 (0.67-0.97)	0.020	0.67 (0.53-0.85)	0.001
Never smoker	0.66	0.002	1.06	0.670	0.57	<0.001	0.89	0.324	0.69	<0.001	0.60	<0.001

	(0.54-0.80)		(0.81-1.40)		(0.45-0.73)		(0.71-1.12)		(0.57-0.84)		(0.47-0.78)	
**Physical activity**												
Less active	1 (Ref.)		1 (Ref.)		1 (Ref.)		1 (Ref.)		1 (Ref.)		1 (Ref.)	
Moderate active	0.74 (0.64-0.86)	<0.001	0.80 (0.65-1.00)	0.045	0.68 (0.55-0.80)	<0.001	0.83 (0.70-0.98)	0.029	0.81 (0.70-0.93)	0.004	0.73 (0.59-0.89)	0.002
Active	0.80 (0.69-0.92)	0.003	0.95 (0.78-1.16)	0.636	0.86 (0.71-1.06)	0.157	0.93 (0.79-1.10)	0.425	0.85 (0.74-0.98)	0.029	0.86 (0.70-1.05)	0.132
**Education level**												
Tertiary level	1 (Ref.)		1 (Ref.)		1 (Ref.)		1 (Ref.)		1 (Ref.)		1 (Ref.)	
Secondary level	0.94 (0.80-1.11)	0.491	0.93 (0.75-1.15)	0.488	1.06 (0.85-1.31)	0.599	1.20 (1.00-1.45)	0.047	0.99 (0.85-1.16)	0.930	1.02 (0.82-1.27)	0.846
Primary level	0.86 (0.74-1.00)	0.054	0.97 (0.79-1.20)	0.804	0.79 (0.64-0.97)	0.026	1.11 (0.94-1.32)	0.218	1.04 (0.90-1.21)	0.598	1.15 (0.94-1.41)	0.181
**Non diabetic**	0.91 (0.80-1.03)	0.141	0.78 (0.65-0.94)	0.008	0.94 (0.79-1.11)	0.468	0.79 (0.69-0.92)	0.002	0.74 (0.65-0.84)	<0.001	0.87 (0.73-1.03)	0.110
**Living alone**	1.16 (0.97-1.38)	0.101	0.89 (0.70-1.14)	0.353	0.78 (0.62-0.98)	0.032	0.88 (0.72-1.07)	0.191	0.96 (0.81-1.14)	0.664	1.15 (0.90-1.47)	0.252

OR, p value and 95% CI for the inadequacy intake of micronutrients as categorical variable according to area. Model has been adjusted by sex, age, smoking habits, physical activity, educational status, diabetic status, living alone, total energy intake and adherence into MedDiet Q-P17.

Table S7. Multivariable logistic regression model for inadequate intake of 3 or more out 6 micronutrients in accordance with criteria by EFSA according to geographical area.

	**≥3 inadequate intake % Prevalence (95% CI)**	**Odds Ratio (95% CI)**	**P value**
**Geographical area**			
**North area**	40.8 (38.2-43.3)	1 (Ref.)	
**Central area**	33.5 (29.1-37.9)	0.59 (0.44-0.80)	0.001
**East area**	36.6 (34.8-38.3)	0.59 (0.50-0.69)	<0.001
**South area**	37.4 (35.1-39.7)	0.59 (0.49-0.71)	<0.001

Results are expressed as OR and 95% CI for the inadequacy intake of ≥3 micronutrients as categorical variable according to geographical area. The model has been adjusted by sex, age, smoking habits, physical activity, educational status, diabetic status, living alone, total energy intake and adherence to MedDiet.
